# Diagnostic Accuracy, Implementation Barriers, and Equity Implications of Teledermatology in Rural Skin Cancer: Scoping Review

**DOI:** 10.2196/77443

**Published:** 2025-12-29

**Authors:** Andres D Parga, Dorothy S Peng, Toan N Vu, Selene M Kizy, Aisha Khan

**Affiliations:** 1 HCA Florida Oak Hill Hospital, Brooksville, FL, United States; 2 David Geffen School of Medicine, University of California, Los Angeles, 10833 Le Conte Avenue, Los Angeles, CA, 90095, United States, 1 9512344591; 3 School of Medicine and Public Health, University of Wisconsin–Madison, Madison, WI, United States; 4 Oakland University William Beaumont School of Medicine, Oakland University, Rochester, MI, United States; 5 Arkansas College of Osteopathic Medicine, Arkansas Colleges of Health Education, Fort Smith, AR, United States

**Keywords:** basal cell carcinoma, confocal microscopy, dermoscopy, disparity, equity, health services research, medical dermatology, melanoma, qualitative research, squamous cell carcinoma

## Abstract

**Background:**

Skin cancer is the most commonly diagnosed malignancy in the United States, with rural populations facing disproportionate delays in diagnosis due to geographic isolation, workforce shortages, and limited access to dermatologic care. These delays contribute to higher rates of late-stage diagnosis and poorer outcomes. Teledermatology has emerged as a promising solution to expand access to dermatologic evaluation and treatment in underserved settings.

**Objective:**

The review aims to evaluate the diagnostic performance, implementation challenges, and equity considerations of teledermatology in the context of rural skin cancer care, and to assess its potential to improve clinical outcomes in underserved populations.

**Methods:**

A comprehensive literature search was conducted across PubMed, Scopus, Web of Science, and Google Scholar to identify studies published between January 2015 and March 2025. Search terms included “teledermatology,” “skin cancer,” “rural health services,” “telemedicine,” “diagnostic accuracy,” and “health disparities.” Studies evaluating diagnostic metrics, time to diagnosis, patient satisfaction, and implementation barriers were included.

**Results:**

Nine key studies spanning various countries and health care settings were included. Diagnostic sensitivity ranged from 41.9% to 100%, and specificity from 46% to 90%, depending on modality and lesion type. Teledermatology consistently reduced time to diagnosis, in some cases by over 75%, and was associated with high patient satisfaction due to increased convenience and reduced travel. Key barriers included technological limitations, inconsistent imaging protocols, and reimbursement variability. Successful implementation was facilitated by standardized workflows, dermoscopy integration, and centralized platforms.

**Conclusions:**

Teledermatology is a viable and effective approach to addressing disparities in rural skin cancer care. It offers diagnostic accuracy comparable to face-to-face evaluations while reducing wait times and improving patient satisfaction. Overcoming technological and systemic barriers is critical to ensuring equitable, long-term integration of teledermatology in rural health systems.

## Introduction

Skin cancer remains the most commonly diagnosed cancer in the United States, with over 5 million cases treated annually and a rising incidence, particularly among rural populations [1]. While early detection is critical for favorable outcomes, especially for aggressive malignancies like melanoma, rural communities often face compounded barriers to timely dermatologic care. These include geographic isolation, insufficient broadband infrastructure, economic disparities, low digital literacy, and a critical shortage of dermatologists. In fact, fewer than 10% of dermatologists practice in rural areas, despite nearly 20% of the US population residing there [1,2]. This imbalance has led to higher rates of late-stage diagnosis, increased morbidity, and poorer survival outcomes in rural regions [1]. Teledermatology, a digital health modality leveraging store-and-forward (SAF) imaging, live-video consultations, and mobile dermoscopy, has emerged as a transformative approach to expanding dermatologic care access in underserved areas. The modality has shown promise not only in reducing time to diagnosis and improving triage efficiency, but also in fostering educational support for primary care providers who are often tasked with managing dermatologic disease in specialist-scarce settings [2,3]. For example, studies have found that mobile teledermoscopy (MTD) enables high diagnostic concordance for skin cancers while minimizing the need for long-distance patient travel [3-5]. Despite its promise, teledermatology’s implementation remains inconsistent, and its integration into rural health care systems is often challenged by technological, regulatory, and infrastructural limitations. These include variable reimbursement policies, lack of standardized imaging protocols, and gaps in digital training among patients and providers [6,7]. Moreover, equity concerns persist, as telehealth’s digital divide may inadvertently exclude older adults, low-income populations, and non-English-speaking patients, groups already at heightened risk for poor skin-cancer outcomes. This review synthesizes the current evidence on the role of teledermatology in rural skin cancer care, with a focus on diagnostic accuracy, implementation barriers, and equity implications. By critically examining recent literature across diverse health care settings, this review aims to inform policy, guide future research, and support equitable access to dermatologic care in geographically and socioeconomically underserved populations.

## Methods

### Study Design

A comprehensive literature search was conducted according to PRISMA-ScR (Preferred Reporting Items for Systematic reviews and Meta-Analyses extension for Scoping Reviews) guidelines (Checklist 1) across 4 major electronic databases: PubMed, Scopus, Web of Science, and Google Scholar. The search included studies published between January 2015 and March 2025 to capture both foundational and contemporary practices in teledermatology. Boolean operators (“AND” or “OR”) were used to combine relevant Medical Subject Headings and keywords, including teledermatology, skin cancer, rural health services, skin neoplasms, telemedicine, remote consultation, diagnostic accuracy, dermoscopy, and health disparities. In addition, reference lists of key articles were manually screened to identify relevant studies not indexed in the databases. We included peer-reviewed studies published in English between January 1, 2015, and March 31, 2025, that (1) evaluated a teledermatology modality (store-and-forward, live video, mobile teledermoscopy, tele-reflectance confocal microscopy [RCM] or cutaneous confocal microscopy, or hybrid) in the context of skin cancer triage, diagnosis, or management, and (2) reported at least one end point relevant to rural care (diagnostic accuracy, time to diagnosis, patient satisfaction, and implementation barriers or facilitators). We excluded editorials, conference abstracts without full data, simulation or algorithm-only studies without clinical end points, pediatric-only studies not focused on cancer, and studies without extractable outcomes.

### Screening and Study Selection

Two reviewers independently screened titles or abstracts and then full texts; disagreements were resolved by a third reviewer. Nine primary studies met inclusion criteria for structured synthesis; additional narrative sources are cited for context.

### Data Extraction

Using a standardized template, we extracted study setting, population, teledermatology modality, outcomes, and key estimates (eg, sensitivity, specificity, time to diagnosis, patient satisfaction, and barriers or facilitators).

## Results

### Overview

**Figure 1.**
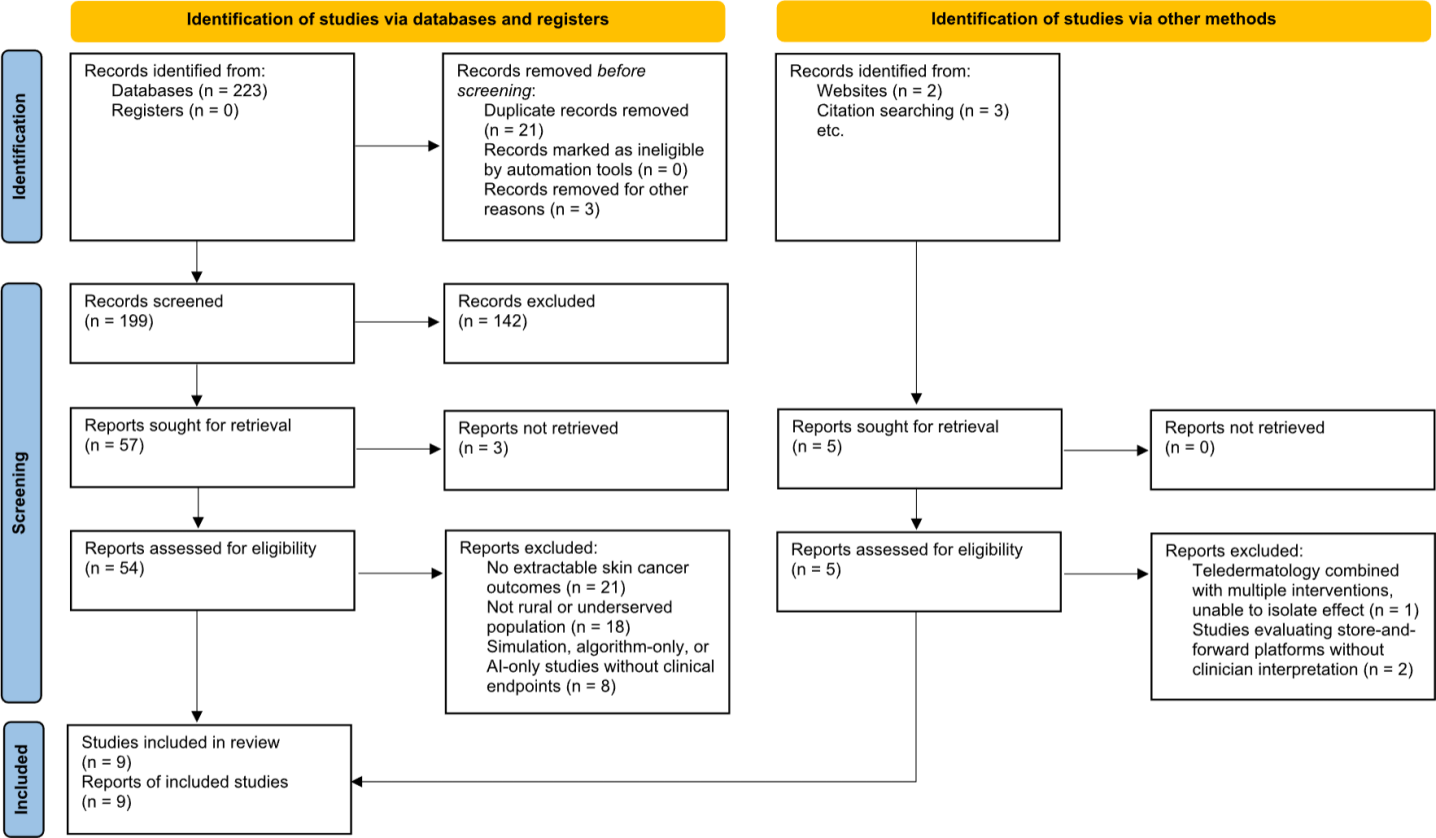
PRISMA (Preferred Reporting Items for Systematic Reviews and Meta-Analyses) flowchart. AI: artificial intelligence.

This literature review synthesizes data from teledermatology studies conducted between 2015 and 2025, evaluating their efficacy in diagnosing skin malignancies among rural populations (Figure 1). Nine key studies provided insights into diagnostic accuracy, efficiency, patient satisfaction, and implementation challenges (Table 1). These included single-country studies from Australia, Spain, the United Kingdom, the United States, Israel, and Italy, as well as 1 multicountry systematic review.

**Table 1.** Diagnostic modalities and their strengths^a^.

Modality	Typical use case	Strengths	Limitations
SAF^b^	Asynchronous triage	Widely available Efficient triage	Image-quality variability No real-time interaction
MTD^c^	Patient- or health care provider–initiated capture	Portable, high patient satisfaction	Requires training Inconsistent image quality
Live-video consults	Real-time specialist evaluation	Interactive Suited for complex decision-making	Scheduling logistics Broadband connectivity needs
RCM^d^	High-risk lesion evaluation	High-resolution cellular imaging Diagnostic depth	High cost Limited access Requires training

a This table compares key teledermatology modalities based on use case, strengths, and limitations. Understanding the differences among these technologies helps inform modality selection for rural implementation strategies.

b SAF: store-and-forward.

c MTD: mobile teledermoscopy.

d RCM: reflectance confocal microscopy.

### Diagnostic Accuracy and Concordance

The diagnostic accuracy of teledermatology for skin cancer was evaluated across the studies reviewed, highlighting its dependence on teledermatology modality, lesion characteristics, and quality of imaging (Table 2). Janda [4] used MTD for lesions, including actinic keratosis and skin cancers such as squamous cell carcinoma and basal cell carcinoma, reporting sensitivity between 41.9% to 79.4% and specificity ranging from 56.2% to 89%. Using SAF dermoscopy, Ferrándiz et al [8] demonstrated higher accuracy, with sensitivity around 90% and specificity approximately 80% for suspicious pigmented and nonpigmented lesions. The systematic review by Chuchu et al [9] covered various modalities, including SAF, live video, and hybrid approaches, presenting robust pooled sensitivity and specificity of 94% and 85%, respectively.

**Table 2.** Regional comparison of teledermatology programs. This table compares teledermatology implementations across 6 regions, highlighting the modalities used, cancer types targeted, diagnostic performance, wait times, key barriers, and programmatic innovations. Data reflect wide variability in technology use and access, with centralized referral systems and artificial intelligence (AI) integration emerging as notable enablers.

Region	Modality used	Skin cancer types	Average sensitivity and specificity (%)	Time to diagnosis	Key barriers	Notable innovations
Australia	MTD^a^ Remote CCM^b^	BCC^c^ SCC^d^ Melanoma AK^e^	Sensitivity: 65‐95 Specificity: 56‐90	Not quantified	Image quality Connectivity issues	Cloud-based RCM^f^ sharing Self-exam empowerment
The United Kingdom	SAF^g^ Live video	Suspected skin cancer Melanoma	Not explicitly quantified	8-13 days	Image quality Patient exclusions	High-quality medical photography Direct discharges
The United States	SAF Telepathology	NMSC^h^ Melanoma	Sensitivity: 84‐95 Specificity: 64‐84	12‐14 days	Training gaps Access gaps for older adults	Telepathology integration EMR^i^ connection
Spain	SAF with dermoscopy	Pigmented and nonpigmented lesions	Sensitivity: approxiamtely 90 Specificity: approximately 80	Reported reduction (not quantified)	Inconsistent infrastructure	Clinical support tools
Chile	SAF	Suspicious skin malignancies	Not specified	7.1‐76 days	Digital divide Long waits	Centralized referral system National telehealth platform
Multicountry	Hybrid SAF Live video	Melanoma SCC BCC	Sensitivity: 46-100 Specificity: 46‐90	Varied (up to 70-d reduction)	Image quality Reimbursement Training gaps	AI^j^ Dermoscopy Provider training

a MTD: mobile teledermoscopy.

b CCM: cutaneous confocal microscopy.

c BCC: basal cell carcinoma.

d SCC: squamous cell carcinoma.

e AK: actinic keratosis.

f RCM: reflectance confocal microscopy.

g SAF: store-and-forward.

h NMSC: nonmelanoma skin cancer

i EMR: electronic medical record.

j AI: artificial intelligence.

Furthermore, sensitivity values ranged from 41.9% to 100%, with many studies reporting sensitivity values as high as approximately 95%. Notably, Janda [4] reported a sensitivity range of 41.9% to 79.4%, and Scope et al [6] reported a sensitivity range of 46% to 74% for melanoma diagnosis when using SAF tele-RCM. Recent studies such as Ho et al [5] reported relatively high sensitivity (89%) but lower specificity (64%) using remote cutaneous confocal microscopy, highlighting variability dependent on imaging modality (Table 3). In addition, a few studies reported specificity as high as 90%, and other studies reported specificity as low as 46%.

**Table 3.** Diagnostic accuracy of teledermatology across studies. Sensitivity and specificity values reported for teledermatology-based skin cancer assessment across various platforms and lesion types. Data highlight variability influenced by modality, lesion characteristics, and image quality.

Study	Modality	Lesion type	Sensitivity (%)	Specificity (%)
Chuchu et al [9]	SAF^a^ Video conference Hybrid	Skin cancer	94.9	84.3
Janda [4]	MTD^b^ SAF	AK^c^ SCC^d^ BCC^e^	41.9‐79.4	56.2‐89.6
Scope et al [6]	Tele-RCM^f^	Melanoma-suspect lesions	46‐74	46‐84
Ho et al [5]	Remote CCM^g^	BCC SCC Melanoma	89	64
Ferrándiz et al [8]	SAF with dermoscopy	Pigmented or nonpigmented	Approximately 90	Approximately 80
Hunt et al [10]	SAF with professional photography	Malignant with premalignant skin lesions	NR^h^	Comparable to biopsy rate
Bruce et al [11]	SAF Video conference	BCC SCC Melanoma	NR	Comparable to in-person
Gómez Arias et al [12]	SAF with dermoscopy	Pigmented lesions, including melanoma	NR	Validated pathologically

a SAF: store-and-forward.

b MTD: mobile teledermoscopy.

c AK: actinic keratosis.

d SCC: squamous cell carcinoma.

e BCC: basal cell carcinoma.

f RCM: reflectance confocal microscopy.

g CCM: cutaneous confocal microscopy.

h NR: not reported.

Teledermatology assessments also showed strong agreement with both histopathologic and in-person clinical diagnoses, with a study reporting histopathology concordance rates (Table 4). Mashoudy et al [7] reported a histopathology concordance range of 67% to 75%, while sensitivity and specificity were reported as approximately 95% and 84%, respectively. Janda [4] also reported a κ value of 0.90 when comparing the diagnostic accuracy of teledermatology with in-person clinical examination. Another study by Hunt et al [10] found that there were no significant differences in biopsy rates and lesion types between teledermatology and face-to-face groups for skin cancer management.

**Table 4.** Types of diagnostic concordance in teledermatology. This table shows how often teledermatology diagnoses matched either biopsy results (the gold standard) or in-person clinical assessments. The results suggest that teledermatology can reliably guide diagnosis and treatment decisions [4,7,10].

Modality	Concordance metric	Value (%)	Agreement summary
SAF^a^, dermoscopy, and telepathology	Histopath concordance^b^	67‐75	Moderate to strong agreement with biopsy-confirmed diagnoses
MTD^c^ and SAF	κ^d^ (teledermatology versus clinical)	90	Excellent agreement with F2F^e^ clinical diagnosis
SAF (photography)	Biopsy rate^f^ teledermatology versus F2F	38.7 versus 50.7 (NS^g^)	Comparable clinical decisions regarding biopsies

a SAF: store-and-forward.

b “Histopathologic concordance” refers to the percentage of teledermatology evaluations that were later confirmed by biopsy.

c MTD: mobile teledermoscopy.

d “κ agreement” measures how closely teledermatology and F2F doctors agreed on a diagnosis, with values near 1.0 indicating excellent consistency.

e F2F: face-to-face

f Biopsy rate comparison shows whether teledermatology resulted in similar clinical actions (like performing a biopsy) compared to traditional visits.

g NS: not significant

### Reduction in Time to Diagnosis and Patient Satisfaction

Teledermatology consistently demonstrated significant improvements in reducing the time to diagnosis (Table 5). Bruce et al [11] and Chuchu et al [9] described marked reductions in wait times, though precise durations were not consistently quantified. Hunt et al [10] found that the median wait time for an appointment with teledermatology was 8 days compared to 13 days for an F2F (face-to-face) visit (*P*<.001) [10]. This was adjusted to 10 days versus 13 days when accounting for postage delays, with a statistically significant difference (*P*=.003). Gomez Arias et al [12] similarly found a reduction from 20.5 to 7.1 days. In addition, Mashoudy et al [7] reported a reduction in wait time from 88.6 days with in-person dermatology to 12.3 days with the use of teledermatology. Jones and Oakley [13] also found that the average response time for advice with teledermatology was 72 hours, suggesting efficient decision-making and triage when using teledermatology. The impact of teledermatology on treatment timelines was also highlighted in a study conducted by Lee et al [14]. This study reported a 2-week reduction in time to treatment with teledermatology, alongside an increase in the amount of lesions treated within 60 days.

**Table 5.** Modality comparison summary.

Modality	Sensitivity or specificity range (%)	Time to diagnosis	Patient satisfaction	Notes
SAF^a^	Sensitivity: 46-94 Specificity: 46-85	8‐20 days	High	Widely used
MTD^b^	Sensitivity: 82‐100 Specificity: 90	Not always stated	Very high	Good for triage
RCM^c^	Sensitivity: 46‐89 Specificity: 46‐64	Not quantified	Mixed	Advanced technology and lower scalability

a SAF: store-and-forward.

b MTD: mobile teledermoscopy.

c RCM: reflectance confocal microscopy.

Regarding patient satisfaction with the teledermatology programs, Ferrándiz et al [8] reported satisfaction rates exceeding 85%, while Hunt et al [10] reported that 88.2% of teledermatology patients were “extremely likely” to recommend teledermatology, in comparison to the 80% of F2F patients who were “extremely likely” to recommend F2F consultations. In addition, Mashoudy et al [7] reported an increased willingness to pay out-of-pocket for teledermatology, highlighting the preference for these services by patients. Although not all studies assessed patient satisfaction explicitly, general reports indicated positive patient experiences associated with teledermatology programs.

### Identified Barriers and Facilitators

Several barriers and facilitators to successful teledermatology implementation were identified. Common barriers included variability in image quality, infrastructure limitations, technological reliability, regulatory differences, geographic disparities, and lack of standardized protocols. In contrast, factors facilitating teledermatology adoption included standardized training, integration with clinical workflows, centralized digital platforms, and clinical support tools. High-quality medical photography, protocolized imaging, and cloud-based platforms were specifically noted as crucial for maintaining diagnostic accuracy and patient management efficiency.

## Discussion

### Principal Findings

Taken together, the included studies indicate that teledermatology can reproduce the clinical decisions of face-to-face care for skin cancer triage when programs prioritize standardized imaging and clear workflows. The main driver of accuracy was not the synchronous or asynchronous format but whether high-quality dermoscopic images and basic capture protocols were in place. Time-to-diagnosis improvements were largest where baseline access was limited, and patient satisfaction tracked with turnaround speed and clarity of follow-up rather than technology brand or platform. Additional reviews and context papers are cited narratively to contextualize implementation and equity considerations but were not part of the structured synthesis.

### Diagnostic Accuracy and Concordance

Specific trends regarding the diagnostic accuracy of teledermatology interventions were found. From the literature review, the sensitivity of teledermatology appears high, underscoring the potential effectiveness of teledermatology in identifying skin cancer correctly and supporting its reliability in diagnosis and management. The ranges in sensitivity may indicate that there is variability across teledermatology interventions for accurately diagnosing various skin cancers. From this review, specificity values were found to be variable as well. Reflected by the variability in sensitivity and specificity, the performance of these teledermatology interventions may vary based on the specific lesion focus. For example, SAF models may underperform during the evaluation of atypical lesions that mimic malignancy or malignant lesions without classic features, as structural detail would be important for diagnosis but may be poorly imaged or lack dermoscopic viewing with SAF. On the other hand, modalities that allow for high-resolution imaging and real-time evaluation and decision-making such as live-video consults, MTD, and RCM may demonstrate higher sensitivity and specificity for these specific lesions. Notably, several studies reported high specificity, particularly when dermoscopy was used, highlighting image quality as a key determinant of diagnostic accuracy. However, these latter modalities have their own limitations in accessibility and cost. Moreover, the sensitivity and specificity values at the lower end of the ranges across these studies may indicate certain barriers in teledermatology, especially in rural areas. These could include inadequate access to reliable technology, lower-quality imaging, and challenges in remotely evaluating and diagnosing different types of skin cancer. Therefore, the performance of teledermatology interventions in diagnosing skin cancer remains multifaceted and important to consider for implementation in rural communities, as it cannot be generalized across all settings.

Although there was variability in the sensitivity and specificity of teledermatology approaches in several studies, it was found that teledermatology produced outcomes that were consistent with conventional F2F consultations and diagnoses. Hunt et al [10] suggested that with teledermatology, biopsy rates and resulting diagnoses are still comparable to conventional care. This demonstrates teledermatology’s potential in achieving the effectiveness of in-person clinical consultations. Given that the findings of this review show a considerable agreement between diagnoses made with teledermatology and those made by skin examinations in a clinical setting, teledermatology may yield accurate diagnostic results comparable to those from conventional consultations.

Based on the reported diagnostic concordance ranges, the clinical accuracy of teledermatology in diagnosing skin cancer lesions can be validated by histopathology. However, only Mashoudy et al reported a histopathology concordance [7]. Thus, the ability to fully evaluate how diagnostic results from teledermatology align with histopathological confirmations remains limited.

To strengthen external validation, future studies should link teledermatology assessments to lesion-level histopathology using unique image IDs and prospective tracking across sites. Multicenter cohorts with pre-registered protocols, blinded reference standards, and standardized dermoscopic capture would enable pooled estimates that are less sensitive to workflow differences. Embedding Quality Assessment of Diagnostic Accuracy Studies-2 (QUADAS-2) at the design stage and reporting per-lesion and per-patient performance will clarify misclassification risk and downstream management impact.

### Reduction in Time to Diagnosis

**Figure 2.**
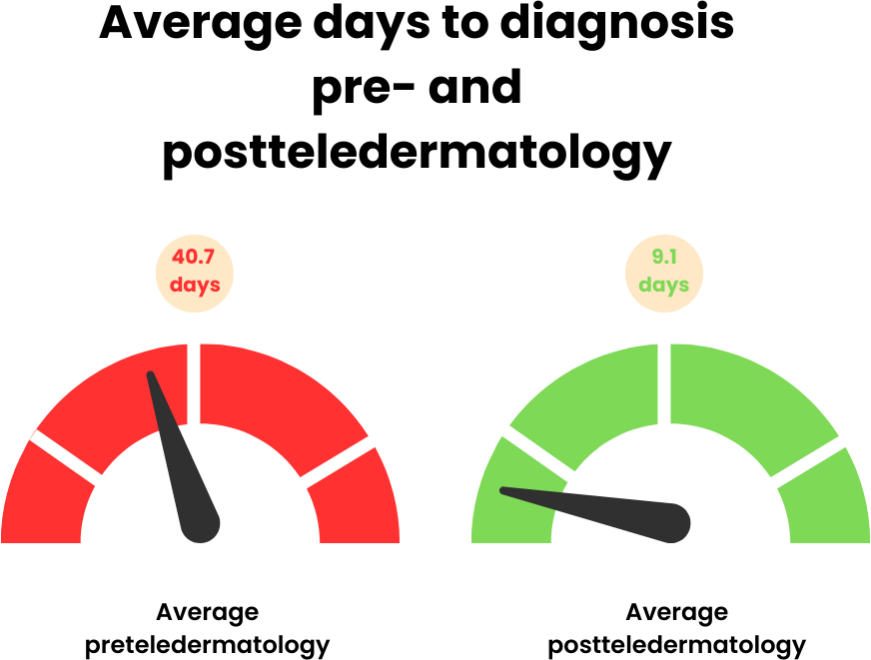
Average time to diagnosis before and after teledermatology implementation. This figure illustrates the average reduction in diagnostic delay across 3 studies: Hunt et al [10], Gomez Arias et al [12], and Mashoudy et al [7]. The average preteledermatology wait time was 40.7 days, which decreased to 9.1 days following implementation of teledermatology services. This represents an approximate 77.7% reduction in time to diagnosis, demonstrating the efficiency of teledermatology in improving access to care in rural and underserved settings. SDs are not shown because the underlying studies report heterogenous summary statistics (medians, IQRs, and means with CIs) and do not provide comparable SD values for pooling.

From the literature review, several studies reported reductions in the time to diagnosis with teledermatology when compared to the time to diagnosis with conventional in-person consultations (Table 5). The results of these studies showcase a trend suggesting that teledermatology contributes to a reduction in time to diagnosis (Figure 2). Furthermore, not all of the studies in this literature review presented this explicit metric. A few of the studies reported reductions in time to diagnosis without quantifying the specific time frames, while other studies did not report this metric at all. Although there was an absence of explicit metrics, such as quantifiable time frames, in the results of some studies, the data collected overall supported teledermatology’s role in reducing the time to diagnosis.

### Patient Satisfaction

Of the current literature reviewed in this work, patient satisfaction with teledermatology interventions remained a high outcome in many of the studies. Various factors influence patient satisfaction with teledermatology, such as convenience, privacy, and the motivational effect of teledermatology on patients to perform self-examinations [14]. These specific drivers of satisfaction are especially important in rural communities, where inadequate access to dermatologic care and geographic barriers are present.

While a majority of these studies expressed high patient satisfaction with teledermatology, Janda [4] presented concerns regarding delayed responses and the impersonal experience of SAF models. On the other hand, MTD was associated with a more positive, favorable experience. This difference in satisfaction based on the specific intervention underscores the importance of informing various teledermatology modalities to improve the patient experience. Moreover, although there were many findings suggesting that teledermatology in rural communities yields positive patient satisfaction, several of the studies did not report on this metric. Not only does this reflect an inconsistency in outcome reporting, but it also affirms the need for further research to examine how different teledermatology interventions in rural settings shape the patient experience.

### Identified Barriers and Facilitators

**Figure 3.**
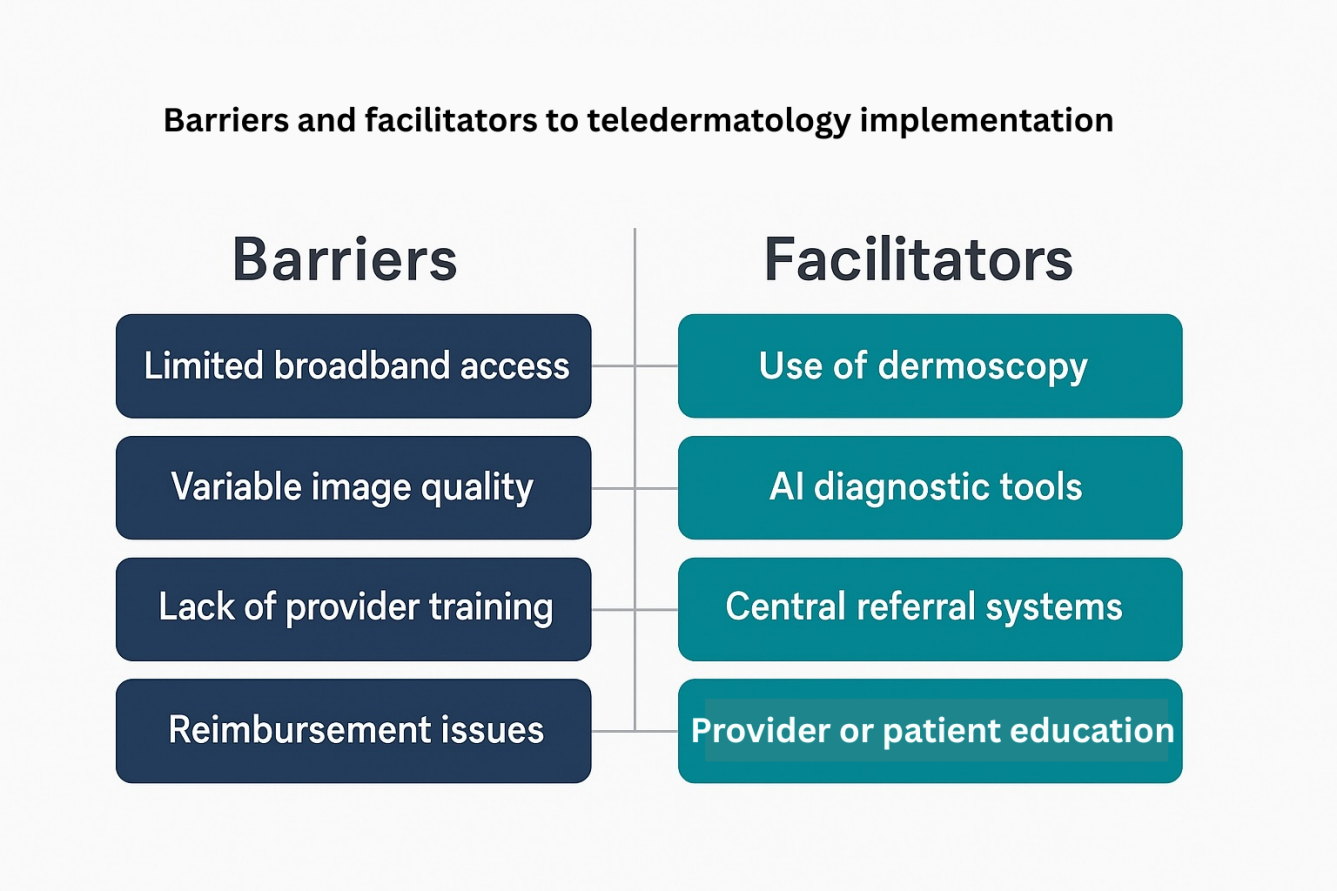
Barriers and facilitators to teledermatology implementation in rural skin cancer care. The framework maps barriers (patient, clinic, system, and policy) to actionable enablers (offline capture, loaner dermatoscopes, navigator support, standardized imaging protocols, reimbursement parity, and rural broadband initiatives) to guide implementation. AI: artificial intelligence.

Teledermatology has a strong potential in the realm of improving dermatological care, especially in underserved and rural regions. One of the most notable benefits of using teledermatology is the reduction in time to diagnosis. Santiago and Lu [15] found that teledermatology can decrease waiting periods, leading to earlier detection and treatment of skin conditions. The study further states that satisfaction within teledermatology is generally high, which can be attributed to factors such as reduced travel requirements for patients, convenience, and timely access to care. However, there are still barriers, particularly in rural settings. Limited access to high-speed internet is a significant challenge, as many rural areas lack the broadband infrastructure to support current telehealth services. There are patients within these regions who may face further difficulties with limited digital literacy and access to smartphones. This hinders the ability to engage on teledermatology platforms across all devices. Health care providers may also find their own set of obstacles in the delivery of teledermatology services, such as varying reimbursement policies and different licensing requirements (Figure 3).

Technology access disparities remain the rate-limiting step in many rural regions. Practical strategies include offline capture with batch upload for low-bandwidth areas; loaner dermatoscopes and imaging kits placed in primary care or community sites; brief, pictogram-based capture training for staff and patients; community health workers or navigators to assist with upload and follow-up; multilingual interfaces and interpreter-integrated workflows; and policy levers (reimbursement parity, interstate licensure compacts, and rural broadband grants) to sustain programs. Publishing open, one-page imaging protocols and checklists can normalize quality across dispersed clinics.

### Synthesis of Findings

Programs that embedded dermoscopy and brief, standardized capture protocols achieved higher specificity than photography-only workflows, regardless of whether care was synchronous or asynchronous. Time-to-diagnosis gains were largest in settings with limited baseline access, reflecting triage efficiencies and direct routing to biopsy or definitive care. Patient satisfaction tracked most strongly with turnaround speed and bidirectional communication rather than the specific platform used. Implementation success depended on program design, centralized platforms, protocolized imaging, and clear escalation rules, more than on any single technology.

### Implications for Rural Programs

The following are the implications for rural programs:

1. Prioritize dermoscopy-enabled, protocolized image capture to improve specificity.
2. Design for speed: ensure rapid review and clear follow-up pathways to drive satisfaction and equity.
3. Centralize intake and routing to minimize delays and unnecessary travel.
4. Budget for enablers (training, image kits, and navigators) rather than only software licenses.

### Future Directions and Policy Priorities

Prospective, multisite cohorts should preregister protocols, standardize dermoscopic capture, and link image-level assessments to blinded, lesion-level histopathology. Equity end points (language, broadband or device access, and older adults) should be prespecified. Policy levers, reimbursement parity, interstate licensure compacts, and rural broadband investment are pivotal for durable scale.

### Limitations of Evidence

This review highlights important trends in the use of teledermatology for rural skin cancer care, but certain limitations should be acknowledged. The studies span a wide range of countries, technologies, and clinical settings, resulting in variability in design, outcome measures, and reporting practices. Some studies did not report complete data on sensitivity, specificity, or patient satisfaction, which limited the ability to make consistent comparisons across interventions. In addition, long-term outcomes such as recurrence rates, treatment adherence, or cost-effectiveness were not commonly assessed. Most of the literature focused on high-income regions, potentially limiting the generalizability of findings to lower-resource rural settings. Nevertheless, the available evidence provides a valuable foundation for guiding future research, implementation efforts, and policy development.

### Conclusions

Teledermatology can reproduce face-to-face decision-making for skin cancer triage in rural settings while substantially shortening time to diagnosis and reducing travel burden. Performance depends less on synchronous versus asynchronous format and more on image standardization (including dermoscopy) and rapid, protocolized workflows. Programs yield the greatest benefit where baseline access is limited, and patient satisfaction tracks with turnaround time and clear follow-up pathways. Heterogeneity in methods and incomplete reporting remain barriers to pooled estimates. Future work should link image-level assessments to histopathology across sites with preregistered protocols and equity endpoints, while policy action on reimbursement, licensure, and rural broadband enables durable scale.

## Supplementary material

10.2196/77443 Checklist 1 PRISMA-ScR checklist.

## Abbreviations

F2F: face-to-face
MTD: mobile teledermoscopy
PRISMA-ScR: Preferred Reporting Items for Systematic reviews and Meta-Analyses extension for Scoping Reviews
QUADAS-2: Quality Assessment of Diagnostic Accuracy Studies-2
RCM: reflectance confocal microscopy
SAF: store-and-forward

## References

[1] Johnson MC, Patel P, Ayers A, Spears KM (2025). Resource management challenges in rural dermatological care: a mapping review. Cureus.

[2] Maddukuri S, Patel J, Lipoff JB (2021). Teledermatology addressing disparities in health care access: a review. Curr Dermatol Rep.

[3] Lee CJ, Boyce A, Chequer de Souza J, Evans R (2024). Store-and-forward (asynchronous) doctor-to-dermatologist non-skin cancer specific teledermatology services in Australia: a scoping review. Australas J Dermatol.

[4] Janda M (2015). Teledermatology: Its use in the detection and management of actinic keratosis. Curr Probl Dermatol.

[5] Ho G, Collgros H, Sinz C (2025). Remote cutaneous confocal microscopy: A multicentric prospective study evaluating diagnostic accuracy for melanoma and keratinocyte carcinoma in tertiary settings. J Am Acad Dermatol.

[6] Scope A, Dusza SW, Pellacani G (2019). Accuracy of tele‐consultation on management decisions of lesions suspect for melanoma using reflectance confocal microscopy as a stand‐alone diagnostic tool. Acad Dermatol Venereol.

[7] Mashoudy KD, Perez SM, Nouri K (2024). From diagnosis to intervention: a review of telemedicine’s role in skin cancer care. Arch Dermatol Res.

[8] Ferrándiz L, Ojeda-Vila T, Corrales A (2017). Internet-based skin cancer screening using clinical images alone or in conjunction with dermoscopic images: a randomized teledermoscopy trial. J Am Acad Dermatol.

[9] Chuchu N, Dinnes J, Takwoingi Y (2018). Teledermatology for diagnosing skin cancer in adults. Cochrane Database Syst Rev.

[10] Hunt WTN, Ali L, Marder H, Sansom JE, de Berker DAR (2020). A service evaluation between 2-week wait (2WW) skin cancer referrals via teledermatology and the standard face-to-face pathway at a teaching hospital. Clin Exp Dermatol.

[11] Bruce AF, Mallow JA, Theeke LA (2018). The use of teledermoscopy in the accurate identification of cancerous skin lesions in the adult population: a systematic review. J Telemed Telecare.

[12] Gómez Arias PJ, Arias Blanco MC, Redondo Sánchez J, Escribano Villanueva F, Vélez García-Nieto AJ (2020). Utilidad y eficiencia de la teledermatoscopia en el manejo del cáncer de piel en atención primaria. Medicina de Familia SEMERGEN.

[13] Jones LK, Oakley A (2023). Store-and-forward teledermatology for assessing skin cancer in 2023: literature review. JMIR Dermatol.

[14] Lee C, Witkowski A, Żychowska M, Ludzik J (2022). The role of mobile teledermoscopy in skin cancer triage and management during the COVID-19 pandemic. IJDVL.

[15] Santiago S, Lu J (2023). Patient satisfaction in teledermatology: an updated review. Curr Dermatol Rep.

